# Plant, fungal, bacterial, and nitrogen interactions in the litter layer of a native Patagonian forest

**DOI:** 10.7717/peerj.4754

**Published:** 2018-05-11

**Authors:** Lucía Vivanco, Nicolás Rascovan, Amy T. Austin

**Affiliations:** 1 Instituto de Fisiología y Ecología Vinculado a la Agricultura (IFEVA), Facultad de Agronomía, Universidad de Buenos Aires, CONICET, Buenos Aires, Argentina; 2 Instituto de Agrobiotecnología Rosario (INDEAR), Santa Fe, Argentina; 3 Instituto de Investigaciones Biotecnológicas (IIB), Universidad Nacional de San Martín, Buenos Aires, Argentina

**Keywords:** Microbial communities, Litter decomposition, *Nothofagus*, Home field advantage, Nitrogen addition, Fungi, Bacteria, Leaf litter, Plant species effects, Temperate forest

## Abstract

Plant–microbial interactions in the litter layer represent one of the most relevant interactions for biogeochemical cycling as litter decomposition is a key first step in carbon and nitrogen turnover. However, our understanding of these interactions in the litter layer remains elusive. In an old-growth mixed *Nothofagus* forest in Patagonia, we studied the effects of single tree species identity and the mixture of three tree species on the fungal and bacterial composition in the litter layer. We also evaluated the effects of nitrogen (N) addition on these plant–microbial interactions. In addition, we compared the magnitude of stimulation of litter decomposition due to home field advantage (HFA, decomposition occurs more rapidly when litter is placed beneath the plant species from which it had been derived than beneath a different plant species) and N addition that we previously demonstrated in this same forest, and used microbial information to interpret these results. Tree species identity had a strong and significant effect on the composition of fungal communities but not on the bacterial community of the litter layer. The microbial composition of the litter layer under the tree species mixture show an averaged contribution of each single tree species. N addition did not erase the plant species footprint on the fungal community, and neither altered the bacterial community. N addition stimulated litter decomposition as much as HFA for certain tree species, but the mechanisms behind N and HFA stimulation may have differed. Our results suggest that stimulation of decomposition from N addition might have occurred due to increased microbial activity without large changes in microbial community composition, while HFA may have resulted principally from plant species’ effects on the litter fungal community. Together, our results suggest that plant–microbial interactions can be an unconsidered driver of litter decomposition in temperate forests.

## Introduction

Plant–microbial interactions are increasingly recognized as important drivers of terrestrial ecosystem functioning (van der Putten et al., 2013). These interactions are particularly important aboveground, where microbes drive decomposition of senescent plant material (litter) that lies on the soil surface. Litter decomposition is a key first step in carbon and nutrient turnover in terrestrial ecosystems (Swift, Heal & Anderson, 1979; Schlesinger & Bernhardt, 2013). The effects of plant species identity on litter decomposition have been extensively studied and have contributed greatly to our understanding of the control of litter quality on carbon and nutrient cycling in terrestrial ecosystems (Pérez-Harguindeguy et al., 2000; Vivanco & Austin, 2006). Nevertheless, we still lack a clear understanding of how plant species affect microbial communities, which function as the decomposing engine in many forest ecosystems.

Plant litter is the main source of carbon, energy and nutrients for heterotrophic microbial communities in the litter layer in terrestrial ecosystems. Plant species vary greatly in the physico-chemical characteristics of litter, such as carbon:nitrogen (C:N) ratio, nitrogen (N) and lignin content, and such, the resource quality that they represent for microbial organisms. In addition, plant species also affect the abiotic and the biotic environment in the litter layer through changes in litter biomass, pH, and their interactions with other organisms like herbivores, symbionts and soil fauna (Eviner, Chapin & Vaughn, 2006). Thus, different plant species may give rise to a distinct microbial community in the litter layer through differences in the nature of their litter, and their interactions with other above- and belowground organisms (Austin et al., 2014). This specificity of plant–microbial interactions might change if the availability of resources changes in the environment. Nitrogen (N) is a key resource that microbes obtain from plant litter and from the surrounding environment. Increases in N availability due to N addition have shown important changes in soil bacterial and fungal communities (Leff et al., 2015). It is unclear however, how these changes alter plant–microbial interactions, and if bacteria and fungi are differentially affected.

In most natural ecosystems, plant species co-occur with other plant species creating mixtures of litter from different plant species in the litter layer. Litter mixtures have shown variable effects on decomposition, from additive effects, where litter mixtures decompose at a rate expected from component litter species incubated separately, to strong antagonistic and synergistic effects (Hättenschwiler, Tiunov & Scheu, 2005). Part of this variation has been attributed to the litter chemistry and nutrient transfer among litter types (Handa et al., 2014; Barbe et al., 2017), and the interaction with soil fauna (Hättenschwiler & Gasser, 2005). Litter mixing can also directionally alter the microbial community in the litter layer (Chapman et al., 2013). Yet, the effects of the natural mixing of litter species on bacterial and fungal communities in undisturbed temperate forest have been rarely assessed.

Disentangling plant species effects from other ecosystem variables like climate and soil resources on microbial communities in natural ecosystems is often difficult, mostly because plant species distribution largely reflect plant species interactions with the environment (Chapin et al., 1987). Experimental attempts to overcome this issue include ‘common garden experiments,’ where different plant species are cultivated in a common environment and their effects are evaluated. This approach, however, complicates the exploration of natural long-term plant–microbial interactions, especially with old-growth tree species which may require long time periods to develop sustained plant–microbial interactions.

Patagonian old-growth temperate forests, located at the southern end of South America, offer the opportunity to study long-term plant–microbial interactions in a natural ecosystem with very low historical and present human impact. These Patagonian forests are co-dominated by three tree species of the genus *Nothofagus* distributed in a well-mixed mosaic (Veblen et al., 1996). Within this mosaic, we identified microsites or ‘tree triangles’ defined by the intersection of three tree-canopies that varied in tree *Nothofagus* species identity. We identified four types of tree-triangles, three monospecific (*Nothofagus dombeyi, N. nervosa,* and *N. obliqua*) and a plurispecific tree triangle (*N. dombeyi, N. nervosa,* and *N. obliqua*), that resulted in an appropriate spatial scale to isolate the effects of tree species identity on microbial communities from other ecosystem factors (Vivanco & Austin, 2008) (Fig. S1). In this forest, we previously demonstrated the existence of home field advantage (HFA) for leaf litter decomposition (Vivanco & Austin, 2008). HFA is the observation that leaf litter decomposes faster beneath plants of the same species (home) than beneath different plant species (away), even for slow decomposing litter. We also showed that N addition disrupted HFA and accelerated litter decomposition (Vivanco & Austin, 2011). Our mechanistic understanding of HFA and N effects on litter decomposition is still lacking, but the nature of these phenomena suggests that particular plant–microbial associations in the litter layer might be central to observed changes in carbon turnover due to plant species identity and resource availability (Austin et al., 2014). Resource availability in natural ecosystems can be greatly altered by human activities occurring in neighbour regions. For example, many temperate forests in the Northern Hemisphere have received chronic and increasing inputs of N since the industrial revolution (Dentener et al., 2006). Studies in unpolluted Patagonian forests increase our understanding of the control of N on ecosystem functioning before human domination of natural systems and potential impact in other parts of the world.

Here we characterized the fungal and bacterial community of the litter layer in the *Nothofagus* tree triangles with and without N addition by amplicon sequencing of the Internal transcribed spacer (ITS) region and the 16s rRNA gene respectively, to evaluate plant species–microbial interactions and their changes due to N addition. We additionally quantified the magnitude of change in litter decomposition due to litter incubation away from the home tree triangle and due to N addition, using data from previous studies (Vivanco & Austin, 2008, 2011). We focused on the following questions: (1) can plant species litter generate specific fungal and bacterial communities in the litter layer? (2) Does exogenous N addition alter or modify microbial communities in the litter layer? (3) What is the magnitude of change in litter decomposition due to HFA and N addition? Results obtained from plant–microbial interactions in the litter layer (questions 1 and 2) allowed us to better interpret changes in decomposition due to HFA and N effects (question 3).

## Materials and Methods

### Study site, experimental design, and sample collection

The study was conducted in a *Nothofagus* mixed forest, in Parque Nacional Lanín, 30 km west from San Martín de los Andes (40°08′S, 71°30′W), Argentina (Permit # 573). Average annual precipitation in this area is 2,300 mm and mean monthly temperatures range from 3 °C in winter (July) to almost 15 °C in summer (January). The overstory vegetation of the study site was entirely dominated by three tree species from the genus *Nothofagus*, which were evenly represented in terms of basal area (Vivanco & Austin, 2008). Under-storey vegetation was dense and monospecific, almost completely dominated by a bamboo species (*Chusquea culeou*). *Nothofagus* is a genus that is only present in the Southern Hemisphere (South America and Oceania). This genus dominates the most part of the Patagonian forests. *Nothofagus* usually form monospecific forests, except for the northern part of Patagonia, where three species of the same genus coexist (*N. dombeyi*, *N. obliqua* and *N. nervosa*) (Veblen et al., 1996). In our study site, there were no other tree species present except for the three *Nothofagus* species studied, and no other tree genera, including gymnosperms, were important species in this forest. Thus, although all species are from the same genus, this site is considered a relatively high tree species diversity naturally encountered in mesic Patagonia.

We identified microsites with different tree species composition within the forest mosaic. These microsites or ‘tree triangles’ were defined by the intersection of three tree-canopies that directly controlled micro-environmental conditions on the litter layer (Vivanco & Austin, 2008). The effective area on the litter layer beneath the tree triangles was 4 m^2^. Mono-specific triangles were composed of single *Nothofagus* species: *N. dombeyi*, *N. obliqua* or *N. nervosa.* Plurispecific triangles were composed three trees of different *Nothofagus* species (*N. dombeyi*, *N. obliqua* and *N. nervosa*; Fig. S1). A total of 10 replicates of each triangle were identified, distributed throughout the 6 ha study site. We combined the tree species identity with a N addition treatment (60 kg N ha^−1^ yr^−1^) (Vivanco & Austin, 2011). We used urea (coated sulphur urea, 39% N) to fertilize five replicates of each *Nothofagus* species triangles for five years. Urea was applied on the litter layer distributed at three times during the year at a dose of 20 kg N ha^−1^ at each time point. To characterize the microbial community in the litter layer, we sampled the litter layer in mid-summer (January) after several years of N addition, harvesting a rectangular area of 100 cm^2^ in the centre of the tree triangle. We maintained the litter layer samples at room temperature till frozen and stored at −20 °C. Leaf litter of *Nothofagus* species differ in physico-chemical characteristics like %N and lignin content, C:N ratio, soluble carbohydrates, polyphenols and tensile strength (Vivanco & Austin, 2008).

### DNA extraction and sequencing

We extracted DNA from the litter layer and pyrosequenced fungal and bacterial genes present to determine the relative abundance of fungal and bacterial species in the litter layer. Litter from each tree triangle was ground with a coffee blender. The coffee blender was carefully washed with distilled water and wiped with ethanol between samples to avoid cross contamination. We used 125 mg of ground litter to extract DNA using the MoBio PowerSoil DNA extraction kit MO BIO Laboratories, Inc. (Carlsbald, CA, USA) following the manufacturer’s instructions. The microbial composition was determined by amplifying the ITS1–ITS2 region (ITS1-Fw: 5′-CTTGGTCATTTAGAGGAAGTAA-3′, ITS2-Rv: 5′-GCTGCGTTCTTCATCGATGC-3′) to study fungal communities and the V4 hyper variable region of the 16s rRNA gene (F563-R802 primers from RDP: http://pyro.cme.msu.edu/pyro/help.jsp) for the bacterial fraction. Amplicons were generated for the five replicates of each single and plurispecific *Nothofagus* tree triangle and then, were pooled (N treatments were treated separately). A total of eight sequencing libraries were created for fungi and eight for bacteria: four *Nothofagus* tree species triangles (three monoespecific, one plurispecific) × two nitrogen treatments (control and N added). This approach integrated in a composite sample all possible members of the microbial community, including rare or less common microbial species, although it did not provide true replicates for statistical ANOVA analysis. For this study, DNA pooling represented a cost-effective alternative, as pyrosequencing is still prohibitively expensive in many regions of the world.

The PCR mix (final volume 25 μl) contained 2.5 μl Fast Start High Fidelity 10× Reaction Buffer (Roche Applied Science, Mannheim, Germany), 5 ng of template DNA, 0.1 μM each primer, 1.25 U Fast Start High Fidelity Enzyme Blend (Roche Applied Science, Mannheim, Germany), and 0.2 mM DNTPs. The PCR conditions were 95 °C for 5 min for initial denaturalization, followed by 95 °C for 45 s, 57 °C for 45 s, 72 °C for 60 s in 25 cycles, and a final elongation step at 72 °C for 4 min. Amplicon libraries were purified and sequenced in Roche 454 GS FLX machine using the Titanium chemistry following manufacturer’s instructions at the INDEAR facility in Rosario, Santa Fe, Argentina. A total of 53,571 filter passed sequences with an average length of 250 bp were obtained for bacteria samples and 99,934 filter passed sequences with sequences with an average length of 291 bp for total fungi. Files in standard flowgram format (sff) were demultiplexed using the sff file tool from Roche. Raw sequencing data is publicly available in the National Center for Biotechnology Information (NCBI) website under the bioproject PRJNA415211 and the sequence read archive (SRA) accession number is SRP120626.

Reads were clustered into operational taxonomic units (OTUs) at ≥97% sequence similarity level using UCLUST (Edgar, 2010) for bacteria and fungi with QIIME software package (Caporaso et al., 2010). The most abundant sequence of each OTU was chosen as the representative sequence and all representative sequences were then aligned using PyNast (Caporaso et al., 2010) for bacteria and Muscle (Edgar, 2004) for fungi using QIIME default parameters in both cases. The alignment was filtered and finally the phylogenetic tree was built using Fast Tree (Price, Dehal & Arkin, 2010). For bacteria, each OTU was taxonomically classified using the RDP classifier (Ribosomal Database Project) and the Green Genes database included in QIIME, with a minimum threshold of 80% and an OTU table was finally built for downstream analyses. OTU tables at 97% were also built for total fungi and taxonomic classification of OTU representative sequences was done using the RDP classifier on a custom-made and parsed version of the EMERENCIA (http://www.emerencia.org/fungalitspipeline.html) and UNITE (https://unite.ut.ee/repository.php) fungal databases.

### Microbial community analysis

To determine the microbial composition for each sample, we filtered singleton sequences out and rarified 100 times each sample with the number of sequences of the sample with the lowest count (7,909 and 2,393 for fungi and bacteria respectively). Rarified OTU tables were used to calculate dissimilarity matrices using the Bray–Curtis method in QIIME (Caporaso et al., 2010) and distance matrices were finally used to perform a non-metric multidimensional scaling (NMDS) analysis. As the interactive effects of plant species identity and N addition could not be analysed parametrically due to the lack of true replication as described previously, we compared the effect of each factor separately using a non-parametric analysis, a one-way analysis of similarity (ANOSIM). To test the effect of plant species identity we performed a one-way ANOSIM with 105 permutations, and we performed a separate one-way ANOSIM with 105 permutations to test the effect of N addition. Multivariate analyses were conducted using PRIMER6 (Primer-E, Plymouth, UK). We also compared the relative abundance of different taxa among plant species identity or N addition with one-way ANOVA, and Tukey test comparisons. *P*-value lower than 0.05 was considered statistically significant.

### Home field advantage and N addition effects on litter decomposition

We quantified the magnitude of change in litter decomposition due to HFA and N addition using data from previous studies (Vivanco & Austin, 2008, 2011). Briefly, those experiments involved reciprocal transplants where leaf litter of single each *Nothofagus* species was placed on the soil in their home location (litter and tree triangle of the same plant species) and away from home (litter and tree triangle of different plant species). Reciprocal transplants were performed with and without N addition, and mass loss was assessed over a year (starting in the summer, in January), and the decomposition constant *k* was estimated. The decomposition experiment occurred in precisely the same tree triangles pertaining to the present study where microbial community composition was evaluated. To compare the magnitude of change due to HFA and N addition we used the decomposition rate observed at home without N addition (*k*_home N(−)_) as the common basis for comparison. That is, to evaluate HFA, we calculated the away affect, which is the difference in decomposition occurring away (*k*_away N(−)_) and at home without N addition (*k*_home N(−)_). To quantify N addition effect, we calculated the differences in decomposition occurring at home with (*k*_away N(+)_) and without N addition (*k*_home N(−)_). We also calculated the interactive of HFA and N addition, comparing decomposition away with N addition (*k*_away N(+)_),with decomposition occurring at home without N addition (*k*_home N(−)_). We used the following equations:

(1) $${\rm{Away \,effect}}:\,{{\left( {{k_{{\rm\, {away \,N(}} - {\rm{)}}}} - {k_{{\rm\,{home \,N(}} - {\rm{)}}}}} \right)} \over {{k_{{\rm\,{home \,N(}} - {\rm{)}}}}}}{\rm{ }} \times 100 \text {%} $$

(2) $${\rm{N \,addition \,effect}}:{{\left( {{k_{{\rm\,{home \,N( + )}}}} - {\rm{ }}{k_{{\rm\,{home \,N(}} - {\rm{)}}}}} \right)} \over {{k_{{\rm\,{home \,N(}} - {\rm{)}}}}}}{\rm{ }} \times 100\text {%} $$

(3) $${\rm{Away \,with \,N \,addition}}:{{\left( {{k_{{\rm\,{away \,N( + )}}}} - {\rm{ }}{k_{{\rm\,{home \,N(}} - {\rm{)}}}}} \right)} \over {{k_{{\rm\,{home \,N(}} - {\rm{)}}}}}}{\rm{ }} \times 100\text {%} $$

## Results

### Microbial community in the litter layer of *Nothofagus* forests in Patagonia

**Figure 1:**
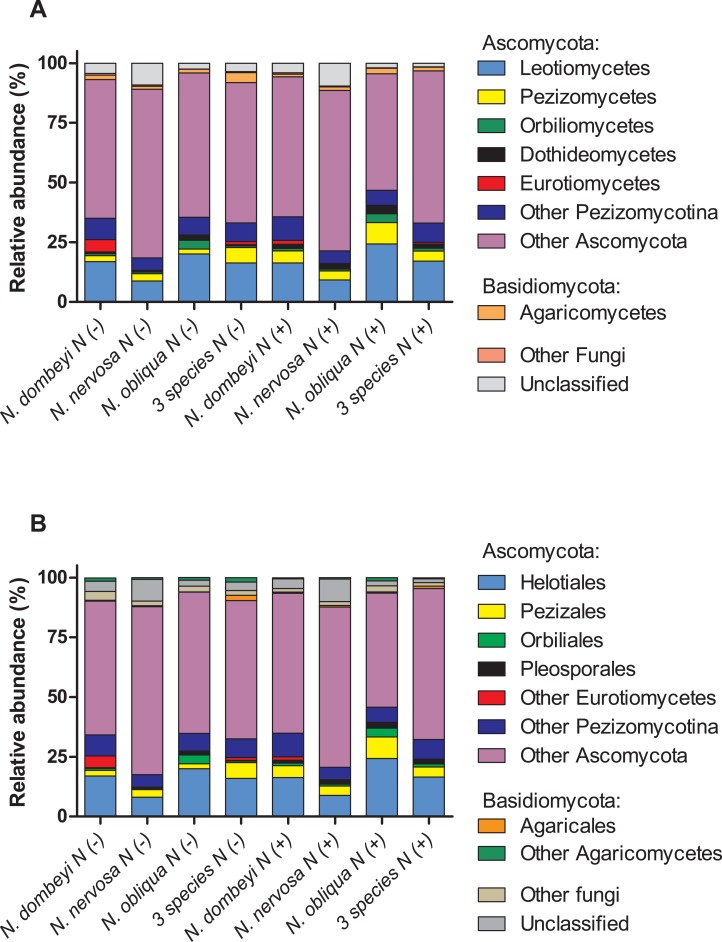
Plant species identity, plant species mixture, and N addition effects on fungal groups in the litter layer of an old growth temperate forest in Patagonia. Relative abundance of (A) fungal classes, and (B) fungal orders in the litter layer of different *Nothofagus* tree species and of three tree species mixture under control (N−) and N-added conditions (N+). Fungal classes with above 1% abundance in the litter layer, and orders above 0.5% abundance are shown.

**Figure 2:**
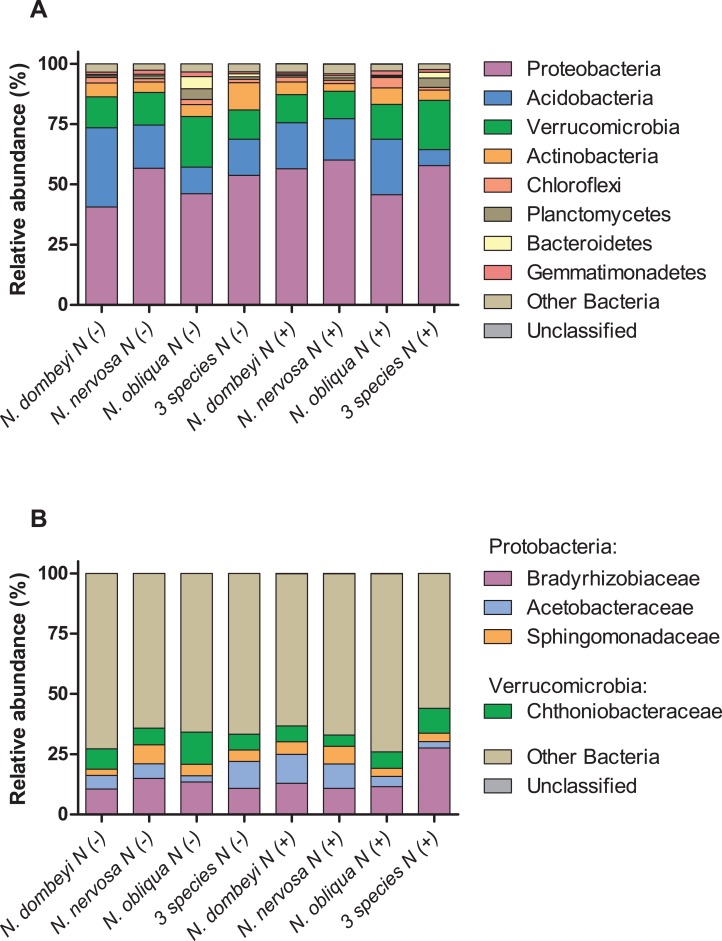
Plant species identity, plant species mixture, and N addition effects on bacterial groups in the litter layer of an old growth temperate forest in Patagonia. Relative abundance of (A) bacterial phyla, and (B) bacterial families in the litter layer of different *Nothofagus* tree species and of three tree species mixture under control (N−) and N-added conditions (N+). Bacterial phyla with above 1% abundance in the litter layer, and families above 5% abundance are shown.

Fungi in the litter layer was dominated by the phyla Ascomycota (93% abundance), with high representation of subphyla Pezizomycotina (33% abundance) whose members mostly belonged to the class Leotiomycetes (16% abundance, Fig. 1A). Heliotiales, Pezizales, Orbiliales, and Pleosporales were the most abundant orders, and together represented 23% of fungal abundance (Fig. 1B). *Dermae* and *Mycoarthris*, from the order Heliotiales, and *Phialea*, from the order Pezizales, were the most abundant genus with 7%, 4%, and 5% of abundance respectively. Members of phyla Basidiomycota were present in very low abundance (2%), and most of them belong to the order Agaricales (Fig. 1B). Bacterial communities in the litter layer were dominated by phyla Alphaproteobacteria, Acidobacteria, Verrucomicrobia with 39%, 18%, and 15% abundance respectively (Fig. 2A). The most abundant families (above 5% abundance) were Bradyrhizobiaceae, Acetobacteraceae, Sphingomonadaceae (all Alphaproteobacteria), and Chthoniobacteraceae (Verrucomicrobia) (Fig. 2B).

### Stronger fungal than bacterial association with tree species in the litter layer

**Figure 3:**
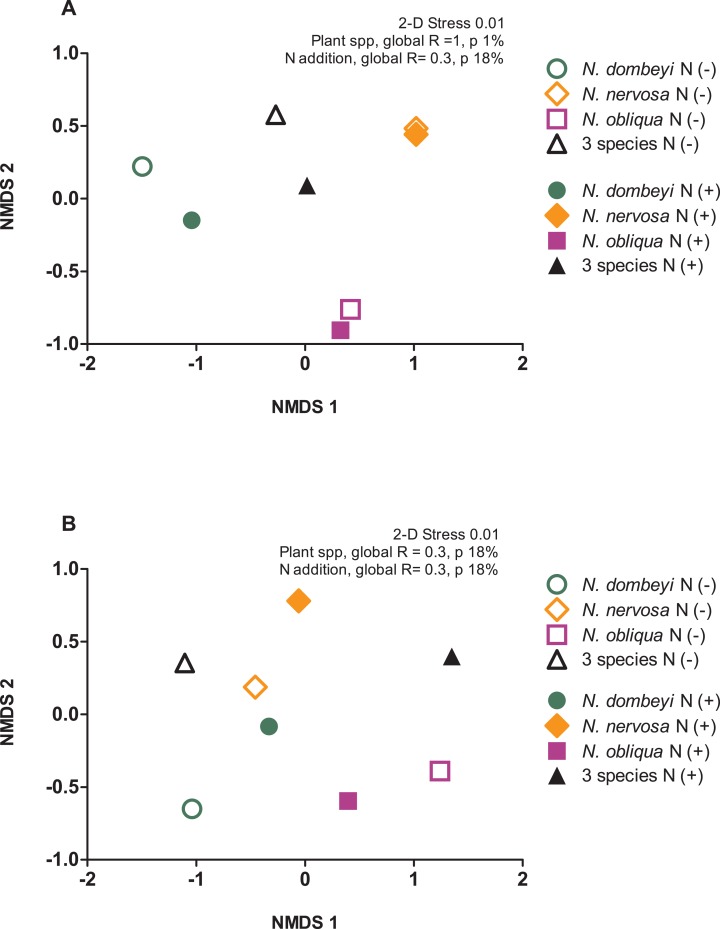
Plant species composition and N addition effects on fungal and bacterial communities in the litter layer in an old growth temperate forest in Patagonia. Nonmetric multidimensional scaling (NMDS) ordination based on Bray–Curtis similarities depicting (A) fungal and (B) bacterial community composition. The shapes of the symbols represent the *Nothofagus* tree species identity, either under control (N−, open symbols) and N-added conditions (N+, closed symbols).

Plant species identity had a significant effect on the fungal communities of the litter layer (ANOSIM, global *R* = 1, *p* = 0.01, Fig. 3A). The relative abundance of the most abundant fungal taxa varied among tree species. The litter layer of *N. nervosa* trees had two times lower abundance of Helotiales, the most abundant order in the litter layer, than under *N. dombeyi* and *N. obliqua* trees (Tukey test, *p* = 0.01, Fig. 1B). *N. nervosa* also had three times higher abundance of unclassified sequences (Tukey test, *p* = 0.006). The litter layer of *N. obliqua* trees had four times higher abundance of order Orbiliales and two times higher abundance of order Pleosporales than *N. dombeyi* and *N. nervosa* trees (Tukey test, *p* = 0.015, 0.029 respectively, Fig. 1B). Finally, the litter layer of *N. dombeyi* trees showed higher abundance of class Eurotiomycetes than *N. nervosa* and *N. obliqua* although differences were not significant (Fig. 1B). In contrast, plant species identity did not have significant effects on the bacterial communities of the litter layer (ANOSIM, global *R* = 0.3, *p* = 0.18, Fig. 3B).

### Predictable microbial composition in the litter layer of tree species mix

The litter layer under the mix of three *Nothofagus* species showed fungal and bacterial communities similar to the average composition of the microbial communities associated to single *Nothofagus* tree species. Fungal communities in the plurispecific tree triangle showed an intermediate position between monospecific tree triangles in NMDS analysis (Fig. 3A), and bacterial communities were similar among tree triangles (ANOSIM, global *R* = 0.3, *p* = 0.18, Fig. 3B).

### Nitrogen addition did not erase plant species footprint on the fungal communities in the litter layer

Nitrogen addition did not significantly alter the fungal and bacterial composition of the litter layer (fungi: ANOSIM, global *R* = −0.135, *p* = 0.8, Fig. 3A; bacteria: ANOSIM, global *R* = −0.021, *p* = 0.42, Fig. 3B).

### The relative size effect of home field advantage and nitrogen addition on litter decomposition depended on plant species identity

**Figure 4:**
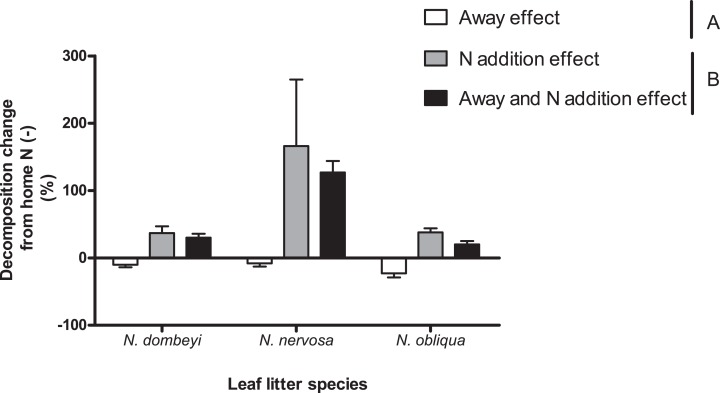
Home field advantage and Nitrogen addition effects on litter decomposition of *Nothofagus* tree species in the Patagonian forest. Values are percent of change in decomposition at a given location compared to decomposition occurring at home without N addition (Home N (−)). Negative values indicate slower decomposition than the reference Home N (−), and it is a way to appreciate HFA (Away effect). Positive values indicate faster decomposition than the reference Home N (−), and it is a way to quantify N stimulation (N addition effect and Away and N addition effect). Letters (A and B) indicate significant differences (*p* >0.05) between Away effect and N addition effect (at home or away). See “Methods” for details.

Nitrogen addition stimulated decomposition of *N. dombeyi* and *N. nervosa* litter more than HFA (Fig. 4). In contrast, N addition and HFA stimulated decomposition of *N. obliqua* litter similarly (∼25%, Fig. 4). Interestingly, N addition stimulated litter decomposition at similar rates when added at home or away from the tree species that originated the litter (Fig. 4). The large and consistently positive effects of N addition on decomposition away from home indicates that N stimulation of decomposition surpassed reductions in decomposition due to away from home incubation. Thus, N addition erased the HFA for litter decomposition.

## Discussion

Here we show that plant species identity differentially affected fungal and bacterial communities in the litter layer in old-growth Patagonian temperate forests. Plant species significantly affected the composition of the fungal community. Moreover, plant-fungal specific associations persisted even when the availability of resources was altered by exogenous N addition. In contrast, plant species identity and N addition did not have large effects on the bacterial composition of the litter layer. Plant species effects on fungal communities together with the observed HFA for litter decomposition were consistent with the idea that specific microbial communities may drive faster litter decomposition of the litter of the same species identity of the trees above. The non-significant effect of N addition on microbial composition indicated that N stimulation of litter decomposition was not necessarily mediated by alterations in the microbial community composition, and suggest instead an increased activity of decomposers due to increased resource availability.

The litter layer is an important reservoir of carbon and nutrients in terrestrial ecosystems, and habitat for many organisms, especially in temperate forests. The microbial composition of the litter layer in the Patagonian mixed forest showed two unique characteristics compared with the litter layer of temperate forests in the northern hemisphere. First, the fungal communities of the Patagonian mixed species forest were entirely dominated with Ascomycota (93%), with very low representation by Basidiomycota (2%). This contrasts strongly with studies demonstrating abundances of 50–70% Ascomycota and 20% Basidiomycota in European and North American forests (Schneider et al., 2012; Voříšková et al., 2014; Hesse et al., 2015; Urbanová, Šnajdr & Baldrian, 2015), and the dominance of Basidiomycota in the soil of most terrestrial ecosystems, including southern temperate forests (Tedersoo et al., 2014). Second, the bacterial communities of the Patagonian mixed species forest had much lower representation of Actinobacteria (5%, forth position on the racking of abundance), whereas in other temperate forests, it has been shown to be the second most abundant bacterial group (15%) after Proteobacteria (Schneider et al., 2012; Hesse et al., 2015; Urbanová, Šnajdr & Baldrian, 2015). *Nothofagus*, the southern beech, is a relict flora of Gondwana continent (Veblen et al., 1996). The biogeographic heritage of *Nothofagus* and other members of the flora and fauna in Patagonia might have contributed to the development of unique microbial community composition. While we cannot rule out differences due to primer biases, the importance of biogeographic patterns of microbial distribution should also be considered when interpreting these patterns (Martiny et al., 2011).

Plant species effects on microbial communities has been established in the rhizosphere and soil (Hartmann et al., 2009) but their effects in the litter layer has received less attention. The most common explanation for plant–microbial specificity is the qualitative and quantitative selective pressure that plant resources exerts on the functioning of the microbial community (Wardle et al., 2004; Fanin, Hättenschwiler & Fromin, 2014). In addition, alternatives to direct effects of litter quality could be plant species alteration of the microclimate, soil resources, and pH (Prescott & Grayston, 2013; Urbanová, Šnajdr & Baldrian, 2015). The *Nothofagus* tree species, however, do not show large differences in terms of their litter chemistry and the environmental conditions created by their canopies. *N. nervosa* and *N. obliqua* were indistinguishable in terms of N and lignin content of their leaf litter (Vivanco & Austin, 2008). Soil carbon, N and phosphate content, humidity, and pH were similar under the influence of the *Nothofagus* tree triangles (Vivanco & Austin, 2008). The way in which the specificity of the fungal community can arise in the litter layer is still open to question and alternatives, beyond those directly due to plant litter effects, need to be considered in order to fully understand the mechanisms behind this association.

A possible way in which plant species may contribute to microbial assembly is through phyllosphere microbes that reach the ground in falling leaves as it was suggested in a recent review (Austin et al., 2014). Plant species harbour different fungal and bacterial communities in the phyllosphere of their green leaves (Kembel & Mueller, 2014; Kembel et al., 2014). The microbial communities present in green leaves may persist during retranslocation of nutrients, senescence and leaf abscission, providing an effective means of dispersal and a substantial advantage for saprophagous microorganisms for the initial colonization of litter. This mechanism may explain the persistence of plant-fungal association even with N addition. Evidence for this mechanism come from recent studies showing that fungal species in the phyllosphere are also found in early decomposed leaf litter of the same tree species (Peršoh, 2013; Voříšková & Baldrian, 2013).

In contrast with changes often reported for microbial communities in the soil (Allison, Hanson & Treseder, 2007; Ramirez, Craine & Fierer, 2012; Cederlund et al., 2014; Leff et al., 2015), exogenous N addition in our study did not alter plant-fungal association in the litter layer and did not affect the bacterial community. The other few studies examining microbial composition in the litter layer did not find effects of N addition on bacterial communities but they found effects on fungal communities (Blackwood et al., 2007; Hesse et al., 2015). The independent effects of plant species and N addition in studies focused on microbial communities have been difficult to disentangle because N addition can also change both plant species composition and litter quality. Some of those studies, however, indicate that tree species identity has a stronger effect than N addition on soil fungal communities (Frey et al., 2004; Blackwood et al., 2007; Tedersoo et al., 2016), suggesting that the nature of the organic matter rather than the total availability of N may impose a stronger control on fungal communities. In contrast, N addition has shown stronger effects than plant community (or litter carbon quality) on soil bacterial communities in grasslands and agroecosystems (Ramirez et al., 2010). The soil environment is quite different than the litter layer, where microbial communities are subject to more variable conditions of humidity, temperature, carbon, and nutrient sources, as well as interactions with other organisms. Taken together, the contrasting responses between litter and soil microbial communities in forest ecosystems may reflect unique controls in each environment. This is important given that most metagenomic studies in terrestrial ecosystems have focused on soil and highlights the need to better understand the specific nature of the plant–microbial interactions in the litter as these interactions are responsible for a key step in carbon cycling with unique controls such as HFA.

Plant species identity and N addition effects on microbial communities in the litter layer provide insights into the nature of plant–microbial interactions in litter decomposition. While plant-fungal association does not necessarily imply decomposer specialization, it is noteworthy that in this undisturbed temperate forest plant species identity had an important and lasting effect on the fungal composition of the litter layer, with a large fraction of unique fungal species associations in each of the three *Nothofagus* species. Future studies are needed to bridge this gap between metagenomic information and microbial function in the environment. These results, together with the finding that fungi, not bacteria, are the main producers of extracellular hydrolytic enzymes in decaying leaf litter in forests (Schneider et al., 2012), suggest that fungal communities might be a more important driver of the persistence of plant–microbial affinity relation affecting litter decomposition in forests. At the same time, N stimulation of the decomposition of *Nothofagus* leaf litter was not reflected in changes in microbial community composition, suggesting that an increase in decomposer activity accelerated litter mass loss with N addition. N addition might have alleviated microbial N limitation imposed by the relatively high C:N ratio of leaf litter (Vivanco & Austin, 2008). Also, N stimulated microbial communities were able to decompose home and away litter at similarly accelerated rates (Fig. 3), suggesting that the advantage to decompose home litter (under no-N added conditions) might arise from different microbial traits to scavenge for litter N.

Microorganisms have traditionally been considered the engine of litter decomposition, with little importance given to their community composition. Here we show that microbial composition is impacted by long-term interactions with plant species that may contribute to HFA for litter decomposition. Plant–microbial controls on the carbon cycle can be particularly important in temperate forests where litter decomposition seems to be driven principally by biotic interactions, as opposed to abiotic processes like photodegradation that dominate decomposition in low rainfall terrestrial ecosystems (Austin & Vivanco, 2006). This study highlights plant–microbial interactions as a currently underappreciated element of the controls on litter decomposition in terrestrial ecosystems, particularly in undisturbed natural forests.

## Supplemental Information

10.7717/peerj.4754/supp-1 Supplemental Information 1 Fig. S1. Tree triangle in a native Patagonian forest. Tree species of the genus *Nothofagus* in a temperate forest in Patagonia, South America. The intersection of the tree canopies (*N. obliqua* on the upper left, *N. nervosa* on the bottom left and *N. dombeyi* on the right) directly control belowground conditions. This "tree triangle" design allows studying plant species effects from the litter layer microbial point of view. Photograph courtesy of Jazmín Vrsalovic.

## Additional Information and Declarations

### Field Study Permissions

The following information was supplied relating to field study approvals (i.e., approving body and any reference numbers): Field experiments were approved by Lanin National Park. Permit # 573.

### Data Availability

The following information was supplied regarding data availability: Data is publicly available in NCBI under the bioproject PRJNA415211 and the SRA accession number is SRP120626.
