# Supplementary material for: Plant, fungal, bacterial, and nitrogen interactions in the litter layer of a native Patagonian forest
Source: PeerJ. 2018 May 11;6:e4754. doi: 10.7717/peerj.4754 (PMC5951145; doi:10.7717/peerj.4754)

S1 Figure. Tree triangle in a native Patagonian forest. Tree species of the genus *Nothofagus* in a temperate forest in Patagonia, South America. The intersection of the tree canopies (*N. obliqua* on the upper left, *N. nervosa* on the bottom left and *N. dombeyi* on the right) directly control belowground conditions. This "tree triangle" design allows studying plant species effects from the litter layer microbial point of view. Photograph courtesy of Jazmín Vrsalovic.

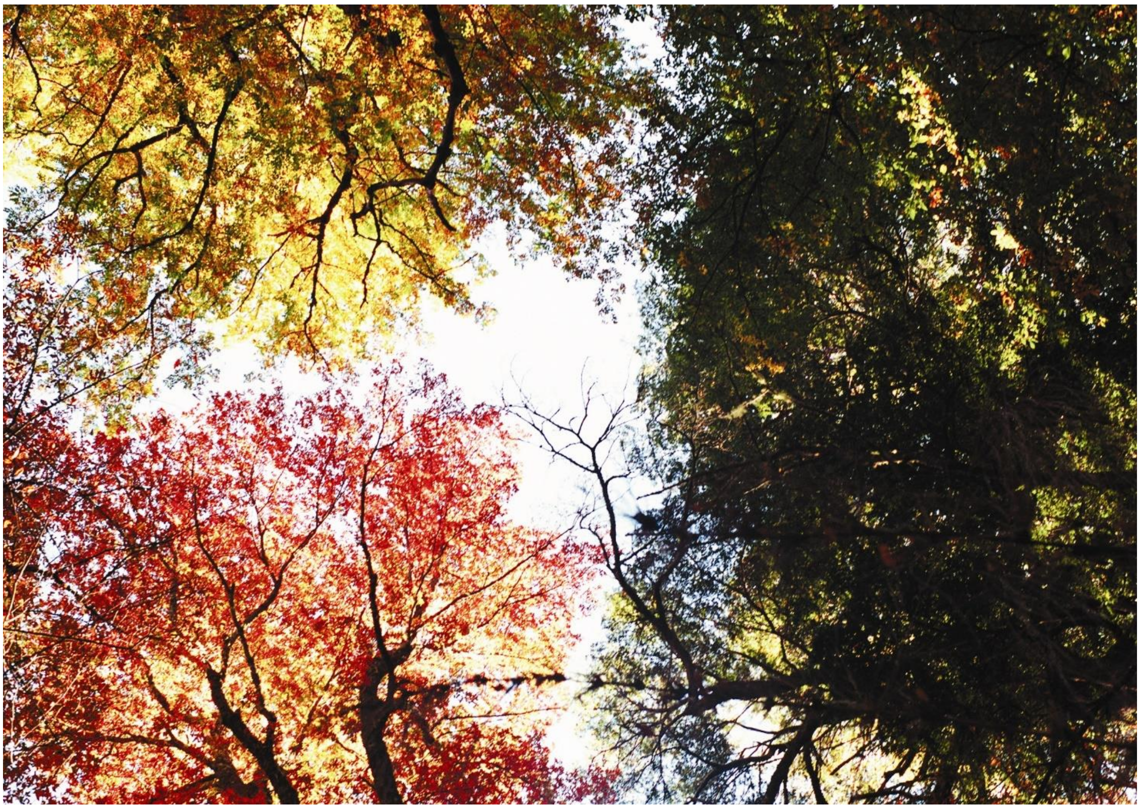

Supplement: Supplemental Information 1 — Tree species of the genus Nothofagus in a temperate forest in Patagonia, South America. The intersection of the tree canopies (N. obliqua on the upper left, N. nervosa on the bottom left and N. dombeyi on the right) directly control belowground conditions. This "tree triangle" design allows studying plant species effects from the litter layer microbial point of view. Photograph courtesy of Jazmín Vrsalovic. [file peerj-06-4754-s001.pdf]
